# Exenatide Use in the Management of Type 2 Diabetes Mellitus

**DOI:** 10.3390/ph3082554

**Published:** 2010-08-11

**Authors:** Angelos Kyriacou, Abu Baker Ahmed

**Affiliations:** Diabetes and Endocrinology Department, Whinney Heys Road, Blackpool Victoria Hospital, FY38NR, UK

**Keywords:** diabetes, exenatide, glycaemic control, incretin mimetics, efficacy, adverse events

## Abstract

Exenatide is a GLP-1 (glucagon-like peptide-1) agonist that has been approved in the UK for use in the management of Type 2 Diabetes Mellitus (T2DM) since 2006. It acts by increasing glucose-induced insulin release and by reducing glucagon secretion postprandially. It therefore increases insulin secretion and reduces glucose levels, especially postprandially. It also reduces gastric emptying and acts centrally to promote satiety. In clinical practice it reduces HbA1c (range; -0.4% to -1.3%), fasting and postprandial blood glucose levels and is the only antidiabetic agent (together with liraglutide; a human GLP-1 analogue) to promote weight loss (range; -1.5 kg to -5.5 kg). It can be used as monotherapy or in combination with metformin and/or sulphonylureas (SU) and/or thiazolinediones (TZD). When compared with insulin it causes similar reductions in HbA1c and glucose levels, but unlike insulin it has the advantage of inducing weight loss. Its main side effect is gastrointestinal (GI) disturbances; nausea is the commonest GI adverse effect, albeit usually mild and transient. Hypoglycaemia is uncommon, especially when used as monotherapy or in combination with metformin. In this review article we scrutinize the currently available evidence for use of exenatide in the management of T2DM.

## 1. Introduction

### 1.1. The incretin-based therapies; biochemical and historical perspective

Gut hormones are well recognized to modulate glucose homeostasis. They are normally secreted from the gut after glucose ingestion, and help regulate the secretion of insulin and glucagon (from the pancreatic islet cells), which in turn regulate hepatic glucose metabolism. These gut hormones were first hypothesized to exist when it was noted that ingested glucose elicits a larger and longer-lasting insulin response compared with intravenous glucose, suggesting that a mechanism existed within the gut that enhances insulin release in response to a meal or oral glucose [1]. This is called the ‘incretin effect’, which is defined as the ratio between insulin secretion during an oral glucose tolerance test and insulin secretion during an intravenous isoglycemic glucose infusion [1]. Two incretin hormones were later identified: GLP-1 and GIP (glucose-dependent insulinotropic peptide). Levels of these hormones were shown to rise rapidly shortly after food intake, and then fall shortly thereafter as a result of rapid inactivation by the enzyme dipeptidyl peptidase-4 (DPP-4) [2]. Of the two hormones, GLP-1 is secreted in greater concentration after nutrient ingestion, and is generally considered more physiologically relevant in humans. The secretion of either of the incretin hormones depends mainly on the quality and quantity of food, with smaller and readily absorbable nutrients stimulating GIP and the converse is true for GLP-1 [3]. Of the two incretin hormones, GLP-1 is secreted mainly from the entero-endocrine cells also known as L-cells of the small intestine (mainly located in the distal small intestine). GLP-1 is produced through post-translational processing of the proglucagon gene. On the other hand, GIP is secreted from another type of enteroendocrine cells, the K-cells (mainly located in the proximal small intestine).

GLP-1 exerts wide array of actions involving an effect on pancreatic islets, as well as extra-pancreatic effects involving actions on hepatocytes, adipocytes, skeletal muscles and central nervous system. In the pancreas it modulates glucose dependent insulin secretion and somatostatin secretion. It also leads to suppression of inappropriate glucagon secretion in the postprandial state. Peripherally, it stimulates glucose uptake and disposal by skeletal and adipose tissues. It also delays gastric emptying and stimulates the satiety centre. GIP-1 also modulates glucose dependent insulin secretion, stimulates glucose uptake and enhances growth, differentiation and neogensis of the beta cells of the pancreas [4]. Microscopically, GLP-1 acts by binding to a specific G-protein coupled receptor which increases intracellular cAMP to potentiate multiple intracellular pathways. 

The incretin effect is well documented to be impaired in patients with T2DM with the primary defect lying at the level of beta cells [5], but impaired GLP-1 secretion from L-cells is also a major contributory factor [6]. The impaired GLP-1 secretion in people with diabetes results in defective glucose-stimulated insulin secretion, reduced glucose clearance and, often, quicker gastric emptying. One major mechanism of impaired incretin effect in T2DM appears to be failure of glucagon suppression following a glucose load [7].

Initial evidence for the efficacy of supplementing incretin activity in patients with T2DM came from short-term experiments in which exogenous, native GLP-1 was administered by continuous intravenous (IV) or subcutaneous (SC) infusion. In these experiments, GLP-1 was shown to reduce blood glucose levels to near normal, restore post-meal insulin secretion and improve beta cell responsiveness to oral glucose [8]. Two facts made initial therapeutic use of GLP-1 difficult. Firstly, GLP-1 has a short half life due to its inhibition by DPP-4 and GLP-4. Secondly, since it is a peptide it cannot be administered orally. In theory a continuous SC or IV infusion would overcome the above problems but such infusions are both expensive and impractical. For these reasons, research has focused on developing compounds that could either mimic the activities of GLP-1 while being less susceptible to DPP-4 inactivation, or that could limit the turnover of endogenous GLP-1 by inhibiting the DPP-4 enzyme. Subsequently, incretin-based therapies, incretin mimetics and DPP-4 inhibitor agents, have been developed. The incretin mimetics, such as exenatide and liraglutide, act by supplementing and/or replacing the activity of endogenous incretins and they have considerably longer half-lives. On the other hand, DPP-4 inhibitors represent the first oral agents targeted at increasing endogenous incretin levels and activity by inhibiting the DPP-4 enzyme.

### 1.2. GLP-1 Analogues (Incretin-mimetics)

The first GLP-1 agonist to be developed was exenatide, whose parent compound was isolated from the saliva of the Gila monster desert lizard (*Heloderma suspectum*). This synthetic GLP-1 agonist was developed and marketed by *Eli Lilly*, as exendine-4 (Exenatide, Byetta®); it shares 53% homology with the GLP-1 amino acid sequence, and binds avidly to the GLP-1 receptors, but is resistant to the action of DPP-4. As a result of this, exanatide has a greatly extended duration of insulinotrophic activity compared to endogenous GLP-1, with a half-life of 2.4 hours *versus* 2–3 minutes, respectively. Through its amino acid sequence homology with GLP-1, it is able to interact with GLP-1 receptors and to mimic all aspects of anti-diabetic activities of GLP-1. Thus, and as a result of stimulation of GLP-1 receptors, exenatide has been found to result in stimulation of the glucose-dependent insulin secretion, suppression of postprandial glucagon secretion, slowing of gastric emptying and reduction of food intake via promotion of satiety [9]. Other actions include restoration of the first phase of insulin secretion and promotion of beta cell proliferation and islet cell neogensis [10]. Long-acting release formulation of exenatide (exenatide LAR) which consist of microspheres containing exenatide and a polymeric matrix has been investigated in phase 2 and 3 trials. It is given as a once weekly SC injection.

The second GLP-1 mimetic is Liraglutide, which is a human GLP-I analogue and is produced by recombinant DNA technology in *Saccharomyces cerevisiae*. Liraglutide is nearly identical to native human GLP-1, with only a single amino acid substitution, and has 97% homology with GLP-1 amino acids sequence.

Below, we examine the place of exenatide in the treatment algorithm of T2DM and scrutinize the evidence that support (or refute) its use in conjunction with or as an alternative to other antidiabetic medications.

## 2. Exenetide Use in the Management of T2DM and Comparison with Other Antidiabetic Agents

Exenatide is currently approved as adjunctive therapy to improve blood glucose control in people with T2DM who are taking metformin, SU, TZD or a combination of metformin and either SU or TZD, and who have not achieved adequate blood glucose control. Exenatide is administered initially 5 µg twice daily injection within 60 minutes of the morning and evening meals and is usually titrated to 10 µg twice daily, after a month’s time or as tolerated.

In the UK, the National Institute for Clinical Excellence (NICE) advocates the use of metformin as a first line agent for the management of T2DM, especially in obese individuals. SU are used as alternatives where metformin is contraindicated, not tolerated or otherwise where a rapid drop in HbA1c is warranted. The combination of metformin plus SU is used as second line; TZD or gliptins can be used as alternatives to SU where SU is contraindicated, not tolerated or otherwise hypoglycaemia might pose a considerable concern. Third line treatment consists of metformin plus SU plus insulin, especially if the patient is markedly hyperglycaemic. Alternatively, a combination of any three of the following agents can be used: metformin, SU, TZD, gliptins or exenatide. Note that exenatide use in conjunction to gliptins is contraindicated. The guidance advices to consider the option of metformin plus SU plus exenatide if the BMI is ≥35 kg/m^2^ in people of European descent who have problems associated with high weight or if BMI <35 kg/m^2^ but insulin is unacceptable because of occupational concerns or weight loss would benefit other co-morbidities [11]. In practice we offer the option of exenatide in the majority of type 2 diabetics with a BMI > 30, who are not achieving glycaemic targets with oral agents, as we feel that weight loss in this patient group would have long-term health benefits. The evidence for use of exenatide with each and combinations of, as well as comparatively to these medications is analyzed below.

### 2.1. Exenatide as an add-on treatment to metformin and/or SU and/or TZD

Three 30-week, placebo-controlled trials, enrolling 1,446 patients, have been conducted to evaluate the safety and efficacy of exenatide in patients with T2DM whose glycaemic control was inadequate with maximally effective dose of metformin alone (n = 336 patients), a SU alone (n = 377 patients), or a combination of SU and metformin (n = 733 patients). Patients were randomized to receive either exenatide (5 µg or 10 µg) or placebo for 30 weeks whilst continuing their existing oral hypoglycaemic agents. The primary end point for all these trials was glycaemic control as assessed by HbA1c. Regardless of the existing oral anti-diabetic agent therapy, exenatide has been shown to result in significant but modest incremental reduction in HbA1c [12,13,14].

The effect of exenatide on metformin treated patients with T2DM was published by DeFronzo *et al.* [12] who studied 334 patients, of whom 272 completed the study. The study showed that the addition of exenatide 5 µg or 10µg to metformin treated type 2 diabetic patients achieved a significant reduction of HbA1c of an average value of 0.4% or 0.8%, respectively, compared to an increase of HbA1c of 0.1% from baseline in the placebo group. Fasting as well as post-prandial blood glucose levels improved with exenatide. There was also added benefit of weight loss of average 3 kg. Mild to moderate nausea was commoner in the exenatide group [12]. 

In another study by Buse *et al.*, the addition of exenatide to a sulphonylurea only treated patients led to a reduction of HbA1c of 0.5% or 0.9% with exenatide 5 or 10 µg, respectively. Fasting blood glucose level decreased in the exenatide group and increased in the placebo group [13].

In the third study by Kendall *et al.* [14], the addition of exenatide 5 µg or 10 µg to the combination of metformin and sulphonylurea was also shown to result in significant reduction in HbA1c (0.6% or 0.8%, respectively) compared to placebo and similar reduction in weight, with average weight loss of 1.6 kg. Fasting blood glucose levels also improved in the exenatide group.

In the above trials, and on an intention to treat (ITT) basis, significantly more of those patients with an HbA1c > 7% at baseline who received exenatide 10 µg in addition to their existing therapy achieved an HbA1c ≤ 7% at 30 weeks [12,13,14]. Long-term extension studies of the exenatide and metformin study [12] and in an open-label 82-week extension of the studies, which involved 314 patients of whom 57% completed full 82 weeks, indicate that long-term exenatide therapy provides incremental reduction of HbA1c of up to 1.3%, when combined with metformin and up to 1.1% in overweight patients; 48% of patients achieved a HbA1c < 7.0% at the end of the study [15].

The concept of combining incretin-mimetics to TZD in patients with T2DM is appealing from a pathophysiological perspective. Glitazones act on insulin resistance and the incretin mimetics target the other facets of metabolic defects of T2DM, namely the abnormalities of defective insulin secretion and inappropriate glucagon secretion [16,17]. Zinman *et al.* [17] conducted a short term study of 16 weeks duration in type 2 diabetics who were sub-optimally controlled with a TZD with or without metformin. Patients were randomized to receive exenatide or placebo in a double blind manner. A change in HbA1c from baseline was the primary endpoint and changes in fasting blood glucose, body weight, and hypoglycemia and GI side effects were the secondary endpoints. Exenatide significantly reduced HbA1c, body weight and fasting blood glucose levels. GI side effects were relatively high (40%). The major limitations of the study are the short duration of the study and SU were not assessed in the study.

### 2.2. Exenatide compared to TZD

In day-to-day practice the dilemma is often whether to use exenatide or a TZD when glycaemic targets are not met by metformin and/or SU. This has been studied by Pinelli *et al.* [18], who evaluated the efficacy and safety of adding exenatide or TZD to oral agents for the management of T2DM. In a metanalysis, the authors evaluated the outcome of 22 publications with 24 month duration or more, which examined the effect of combination of either exenatide or a TZD to other oral antidiabetic agents. The collective endpoints were effects on HbA1c, proportion of subjects reaching target HbA1c of <7%, mean changes in fasting plasma glucose, body weight, and finally the occurrence of severe hypoglycemia, and GI side effects. Both agents were efficacious in improving blood glucose control, but TZD resulted in greater reduction in HbA1c (-0.8%) than exenatide (-0.6%), albeit at the expense of more weight gain; exenatide failed to achieve a decrease in fasting plasma glucose but had the advantage of weight loss. Both agents were associated with an increase in the incidence of mild or moderate hypoglycemia, though more GI side effects in the form of nausea, vomiting and diarrhoea occurred in the exenatide group.

To summarize, incretin mimetics could be a useful combination with TZD, rather than a substitute for TZD, as the current evidence does not support a superior effect of incretin mimetics over TZD.

### 2.3. Exenatide compared to gliptins

The gliptins are oral antidiabetics that act as DPP-4 inhibitors; sitagliptin, vildagliptin and saxagliptin are currently available in the EU market. They cause modest reductions in HbA1c and are weight neutral. 

A recently published randomized double-blind cross-over study compared exenatide with sitagliptin in a head to head study, among type 2 diabetics already receiving metformin. Two hours postprandial glucose, insulin and glucagon secretion, gastric emptying (assessed by acetaminophen absorption) and caloric intake were used as the study endpoint [19]. The study showed that exenatide is superior to sitagliptin in reducing postprandial glucose excursions, reducing insulinogenic index of insulin secretion and reducing glucagon secretion. Exenatide was also superior in reducing postprandial triglyceride concentration, caloric intake and slowing gastric emptying. Both agents were equally effective in reducing the net fasting blood glucose level. The main study limitation was its short duration. In view of the higher cost of exenatide (in the UK exenatide 10 µg bid costs £65 per month compared to £35 per month for sitagliptin) a comparison study was done with sitagliptin from a financial perspective; exenatide was associated with less total medical costs (difference of $655), despite having higher diabetes-related costs [20]. 

### 2.4. Exenatide compared with insulin

A common theme in the diabetic clinic is a question of whether to use exenatide or insulin when a third line agent is needed to achieve glycaemic targets in a patient that has already tried various combinations of oral antidiabetics (e.g. metformin and/or SU and/or TZD). The effect of exenatide on blood glucose control and body weight was compared with that of insulin glargine in a 26-week, open-label, randomized controlled study [21,22]. In these studies, enrolled subjects continued their prior regime with metformin and SU, but then they were randomly assigned to receive additional treatment with either exenatide or insulin glargine. Both exenatide and insulin glargine equally improved blood glucose control, with no difference in HbA1c (-1.25% and -1.26%, respectively). Exenatide reduced postprandial glucose excursions more than insulin glargine, while insulin glargine reduced fasting blood glucose levels more than exenatide. Body weight decreased by 2.3 kg with exenatide and increased by 1.8 kg with insulin glargine (difference, -4.1 kg [CI, -4.6 to -3.5 kg]). Rates of symptomatic hypoglycemia were similar, but nocturnal hypoglycemia occurred less frequently with exenatide. Another retrospective study comparing exenatide and insulin glargine showed reduced risk of glycaemic variability with exenatide [23]. These two treatment modalities were also compared in terms of beta cell function, glycaemic control and body weight. Exenatide use was associated with significantly improved beta cell function (as assessed with a glucose- and arginine-stimulated C-peptide test). Similarly to the aforementioned studies, exenatide also caused significant weight loss (difference -4.6 kg), but similar HbA1c compared to glargine (-0.8 *versus* -0.7, respectively) [24]. A randomized, open-label, 24-week trial, comparing biphasic insulin aspart 70/30 od or bid against exenatide (on a background of metformin and SU), showed that glycaemic control was better with biphasic insulin (in terms of HbA1c and higher proportion of patients reaching target HbA1c of <7% or <6.5%); however, insulin use was associated with higher risk of hypoglycaemia and weight gain [25].

In a review of phase-III human clinical trials and post hoc completer analysis exenatide therapy was confirmed to achieve similar improvement in glycemic control to that achieved by once daily insulin glargine or twice daily isophane insulin [26]. The observed side effect profile consisted mainly of mild to moderate nausea, and rates of hypoglycemia were comparable to that of placebo (when combined with metformin) and to insulin comparator (when combined with metformin & SU). In the same analysis, the use of exenatide with metformin or SU was associated with significant improvement of health related quality of life (HR-QOL). Furthermore, cost-effectiveness analysis also showed that exenatide is comparable to insulin glargine, glibenclamide and pioglitazone when combined with metformin. In a post hoc analysis of pooled data from two other studies, the use of exenatide was compared with insulin analogues (either insulin glargine or biphasic insulin aspart) with changes in weight as the main endpoint; the use of exenatide over 6 month period was associated with an average weight loss of 3 kg whilst patients on insulin gained weight [27].

### 2.5. Exenatide as an add-on treatment to insulin

A problem that is often encountered is a patient that is already on insulin and has poor glycaemic control, either due to problems with hypoglycaemia or weight gain with increasing doses of insulin, or simply due to failure of hefty doses of insulin to reduce the hyperglycaemia. One treatment option is to add a TZD, if this has not already been tried [11]. However, another exciting (but currently unlicensed) option is the addition of exenatide to insulin treatment. This was explored by several investigators. A retrospective study by Viswanathan *et al.* [28] examined the addition of exenatide to a group of 52 subjects with T2DM who were on insulin plus OHA. There was no significant improvement in HbA1c, but the reduction in weight and in total insulin daily requirement was significantly different after 26 weeks of therapy. Of those using exenatide, there was high dropout rate. 

In another prospective study, Govindan *et al.* [29] studied patients with insulin treated diabetes who failed to achieve the HbA1c target and who also had problems with progressive weight gain. At the end of the study, the improvement in HbA1c was not significant, but patients were able to reduce the total daily dose of insulin, and they also managed to lose weight. The incidence of severe hypoglycemia was unchanged by the addition of exenatide. 

In a retrospective study, the use of insulin and exenatide was assessed [30]. It showed weight loss of at least 1 kg in 78% of patients and a drop in HbA1c from 8.1% to 7.2% at 8 weeks. Mild hypoglycaemia occurred in only three patients. The study by Davis *et al.* [31] has suggested that patients with T2DM who have longer diabetes duration and those who are taking higher doses of insulin therapy may experience deterioration of their control if they substitute exenatide for insulin.

A further retrospective study of exenatide addition to insulin has also shown significant improvements in HbA1c and weight [32]. The mean total insulin daily dose was reduced in all patients in 6 and 12 months; when the total insulin dose was stratified between basal and prandial insulin, the reduction in insulin dose was significant for the prandial group only. This suggests that on introducing exenatide in addition to insulin we should perhaps be reducing the prandial insulin dose more than the basal dose.

To summarize, exenatide may prove to be an attractive agent to be used in insulin treated type 2 diabetic patients who are not achieving the adequate level of control. Benefits include in addition to perceived improved glycaemic control, a favorable impact on weight. Also, the potential of significant reduction in total insulin daily requirement and the prospect of stopping insulin all together are other benefits, but the preliminary evidence suggests that this may be confined to patients who have residual beta cell function. More research is certainly required for more rigid recommendations to be produced regarding if and when insulin should be concurrently used with exenatide, and at what doses. As exenatide is not currently licensed to be used in conjunction to insulin, this has to be relayed to the patient concerned, and consent obtained prior to any trial of combination therapy.

### 2.6. Exenatide compared to exenatide LAR

Exenatide LAR is not currently approved, but it is likely that it will become available in the near future [it is currently being assessed for approval by the US Food and Drug Administration (FDA)]. Although data are limited, current evidence indicates that exenatide LAR is an effective and safe antidiabetic agent [33,34]. A 15 week trial compared the effect of exenatide LAR with placebo in patients with suboptimal blood glucose control despite diet, exercise and metformin [33]. In this trial, exenatide LAR resulted in significant reduction in HbA1c of 1.7% and improvement of fasting blood glucose levels. A second trial compared the effect of exenatide LAR on blood glucose control with that of exenatide twice daily; at the end of 30 weeks, HbA1c decreased by 1.9% in exenatide LAR treated patients, compared to 1.5% in patients receiving exenatide twice daily. Treatment with exenatide LAR also resulted in significant lowering of fasting plasma glucose compared to exenatide. Both treatment modalities caused similar weight loss. Nausea was less common in the exenatide LAR group and no severe hypoglycaemia episodes were reported on either treatment arm [34]. Furthermore, exenatide LAR would be a real breakthrough in this area, as a one weekly injection will greatly boost compliance.

### 2.7. Exenatide compared to liraglutide

At present, liraglutide is the only other GLP-1 agonist that is approved for use in the management of T2DM. Exenatide has been compared with liraglutide in the 26-week LEAD 6 trial, involving 464 patients with T2DM treated with maximally toletated dose of metformin and/or SU [35]. Patients were randomised to treatment with either liraglutide 1.8 mg once daily or exenatide 10 µg twice daily for 26 weeks. HbA1c was reduced by 1.12% with liraglutide, which was significantly greater than the reduction with exenatide (0.79%). Fasting blood glucose levels improved with both treatments, although lower levels were achieved with liraglutide compared to exenatide. Mean weight loss was similar (3.24 *vs.* 2.87 kg, respectively). Liraglutide is however more expensive than exenatide and exenatide remains the first line incretin-mimetic treatment at present, given the large volume of positive evidence attached to it. Nevertheless, it is worth noting that liraglutide has the advantage of once daily subcutaneous injections which can be appealing to some of our needlephobic patients.

### 2.8. Exenatide as monotherapy

There is some preliminary evidence to suggest that monotherapy with exenatide may be as effective as a combination of exenatide with oral antidiabetic agents. In a study by Nelson *et al.* [36] who conducted a randomized double-blind trial comparing the use of exenatide 10 µg with placebo, followed by an open label arm of exenatide alone or exenatide with metformin; exenatide 10 µg twice daily as monotherapy was more effective than placebo in achieving the study endpoints (HbA1c and fasting blood glucose). On the other hand, exenatide was as equally effective as monotherapy as in combination therapy with metformin. In another multi-centre, placebo-controlled study by Moretto *et al.*, exenatide use in type 2 diabetics, naïve to treatment with other antidiabetic medicines, was associated with improvements in HbA1c, fasting and postprandial glucose, weight and beta-cell function [37]. 

Indeed, since November 2009 exenatide has received a monotherapy indication from the US Food and Drug Administration (FDA), indicating that it can be used safely and effectively as a single diabetes agent in conjunction with dietary measures and exercise. 

## 3. Adverse Events

The most common adverse effects observed with exenatide therapy [10,12,13,14,15] were GI, with nausea being the commonest adverse effect, occurring in one third to half of exenatide treated patients; vomiting and diarrhea were less common (12-17%). Although these side effects are common, the frequency and severity decreased as treatment continued. Our experience with exenatide is that advice to stop eating as soon as the patient experiences a feeling of ‘fullness’ decreases the risk or the severity of nausea. We generally suggest dietetic input to all our patients initiated on exenatide to ensure dietary interventions remain in place once on exenatide, but also for advice on how to avoid GI side effects with exenatide. Case reports have suggested a link between exenatide and acute pancreatitis [38]. However, when data from a large US commercial insurance company were used to assess the risk of acute pancreatitis with exenatide or sitagliptin against metformin or glyburide, no significant difference was observed [39]. 

The incidence of mild to moderate hypoglycemia was less common when exenatide was used as monotherapy (5%); however, when used in combination with other anti-diabetic agents the incidence of mild to moderate hypoglycaemia was 5% with metformin and up to 36% with a SU. Thus, when combining exenatide with a SU, a reduction in the SU dose should be considered.

The effect of renal impairment on the pharmacokinetics of exenatide was also studied in 31 patients, of whom only one had T2DM [40]. Exenatide was well tolerated in those with mild or moderate renal impairment (Creatinine Clearance > 50 mL/min), but not in subjects with end-stage renal disease (ESRD) needing haemodialysis. Simulations of exenatide plasma levels suggested a trend towards poor tolerability at the lowest available therapeutic dose, for the ESRD patient group. 

There is no evidence that exenatide adversely affects any of the other diabetic microvascular complications, although there is a case report were the introduction of exenatide caused a dramatic drop in HbA1c and a subsequent deterioration of underlying diabetic retinopathy [41]. 

## 4. Beyond Glycaemic and Weight Control; Effects on the Cardiometabolic Syndrome

Beyond their effects on glycaemic control and body weight exenatide has demonstrated additional novel features including positive effects on beta cells, as well as an improvement in lipid profile and blood pressure. The current evidence demonstrated that incretin mimetics improve some parameters of beta cell function during treatment. Although beta cell function cannot be measured directly in human subjects, indices of beta cell mass and function can be estimated indirectly by assessing some markers such as Homeostasis Model of Assessment: Beta-cell function (HOMA-B), the proinsulin to insulin ratio and the arginine stimulated C-peptide. Exenatide treatment has been shown to improve beta cell function; the HOMA-B value increased from baseline by 32% in exenatide treated patients following 24 weeks treatment compared to only 6% for placebo (p = 0.02) [37]. Following one year treatment with exenatide, the arginine stimulated C-peptide test showed a 2.19-fold increase from baseline compared to only 0.3-fold increase for insulin glargine (p < 0.0001) [42]. After 30 weeks of exenatide therapy, insulin secretion rate were shown to increase by up to 72%. Whether the improvement observed in clinical studies are the direct effects of GLP-1 agonists on beta-cell function or is a result of improved blood glucose control remains to be elucidated.

Studies have suggested cardio-protective activities of GLP-1 agonists, although long-term studies to prove cardiovascular benefit are necessary in order to make firm conclusions on that. Current evidence has shown positive effects of exenatide on surrogate cardiovascular parameters such as systolic blood pressure, triglycerides, endothelial function and brain natriuretic peptide. Exenatide studies have demonstrated a marked improvement of lipid profile, with substantial reduction in apolipoprotein B (ApoB) (-5.2 mg/dL), triglycerides (-7.3 mg/dL), and an increase in high density lipoprotein cholesterol (+4.5 mg/dL), together with small reductions in total cholesterol and low density lipoprotein cholesterol [15]. In addition, exenatide appears to be associated with substantial improvement in systolic blood pressure [15]. This improvement of lipid profile and blood pressure may be partly related to weight loss experienced with exenatide therapy. An open-ended, open-label trial was done to assess the effects of three or more years of exenatide use. Along with sustained reductions in HbA1c and weight, there were significant reductions in alanine aminotransferase (ALT) [among patients with elevated ALT at baseline], aspartate aminotransferase (AST), blood pressure and an improvement in the HOMA-B score. Subset analysis showed modest but significant reductions in LDL, total cholesterol and triglycerides and increase in HDL [43]. The improvement in hepatic biomarkers is of note; research into the potential beneficial effects of exenatide on conditions such as Non-Alcoholic Steatohepatitis (NASH) is currently in prorgress.

Although this review article mainly relates to the use of exenatide in T2DM, we feel it is imperative to mention three more potential thrilling avenues for use of GLP-1 agonists:

(1). Pre-diabetes state; the DECODE study showed an increased cardiovascular disease (CVD)-related morbidity and mortality in the pre-diabetes period [44]; by the time the diagnosis of T2DM is reached by existing diagnostic criteria, a large proportion of these patients have established CVD due to a detrimental effect of hyperglycaemia on the vascular endothelium [45]. As reduced GLP-1 response to food may contribute to the development of T2DM, it remains unclear whether intervention at this stage by GLP-1 agonists would delay or cease the progression to T2DM. 

(2). ‘Stress-diabetes’ in the peri-operative and hospital inpatient setting; GLP-1 agonists can be tried in these situations as they counteract the effect of ‘stress hormones’ (steroids and glucagon) and in view of the low incidence of hypoglycaemia. They are especially beneficial in those with pre-diabetes or in T2DM treated with oral antidiabetic medications [45]. However, more research is needed to guide clinicians into the exact place of GLP-1 agonists in these settings. 

(3). Steroid-induced diabetes; GLP-1 agonists counteract the inhibitory effects of steroids (such as dexamethasone) on the beta cell secretion of insulin. This effect may relate partly on the action of GLP-1 agonists on a transcription factor, called PDX-1 (pancreatic and duodenal homeobox-1) [46]. 

## 5. Conclusions

Exenatide appears to be a promising agent in the multitude of drugs used in the management of T2DM. Perhaps the most significant beneficial effect of exenatide is the glucose dependent insulin secretion, which probably mimics the physiological post-prandial insulin secretion. It is therefore associated with low rates of hypoglycaemia. In addition to this, and probably more significant to most of our type 2 diabetic patients exenatide will provoke significant weight loss. Its main disadvantage is that it cannot be taken orally. However; it holds great potential to tackle the long lasting issue of secondary failure to oral hypoglycaemic agents. Its effects on some of the core pathophysiological defects, its positive effect on beta cell mass and function and the parameters of the metabolic syndrome holds great promise; long-term cardiovascular outcome studies are desperately awaited in that respect. Exenatide appears to be an attractive combination with most of the existing oral agents (metformin and/or SU and/or TZD); its use with TZD and possibly with (or more often as an alternative to) insulin may hold specific advantages. 
